# Kinetic Curve Type Assessment for Classification of Breast Lesions Using Dynamic Contrast-Enhanced MR Imaging

**DOI:** 10.1371/journal.pone.0152827

**Published:** 2016-04-07

**Authors:** Shih-Neng Yang, Fang-Jing Li, Jun-Ming Chen, Geoffrey Zhang, Yen-Hsiu Liao, Tzung-Chi Huang

**Affiliations:** 1 Department of Biomedical Imaging and Radiological Science, China Medical University, Taichung City, Taiwan; 2 Department of Radiation Oncology, China Medical University Hospital, Taichung City, Taiwan; 3 Department of Radiation Oncology, Tri-Service General Hospital, Taipei City, Taiwan; 4 Department of Radiology, China Medical University Hospital, Taichung City, Taiwan; 5 Department of Radiation Oncology, Moffitt Cancer Center, Tampa, Florida, United States of America; 6 Department of Bioinformatics and Medical Engineering, Asia University, Taichung City, Taiwan; Le Fe Health Research Institute, SPAIN

## Abstract

**Objective:**

The aim of this study was to employ a kinetic model with dynamic contrast enhancement-magnetic resonance imaging to develop an approach that can efficiently distinguish malignant from benign lesions.

**Materials and Methods:**

A total of 43 patients with 46 lesions who underwent breast dynamic contrast enhancement-magnetic resonance imaging were included in this retrospective study. The distribution of malignant to benign lesions was 31/15 based on histological results. This study integrated a single-compartment kinetic model and dynamic contrast enhancement-magnetic resonance imaging to generate a kinetic modeling curve for improving the accuracy of diagnosis of breast lesions. Kinetic modeling curves of all different lesions were analyzed by three experienced radiologists and classified into one of three given types. Receiver operating characteristic and Kappa statistics were used for the qualitative method. The findings of the three radiologists based on the time-signal intensity curve and the kinetic curve were compared.

**Results:**

An average sensitivity of 82%, a specificity of 65%, an area under the receiver operating characteristic curve of 0.76, and a positive predictive value of 82% and negative predictive value of 63% was shown with the kinetic model (p = 0.017, 0.052, 0.068), as compared to an average sensitivity of 80%, a specificity of 55%, an area under the receiver operating characteristic of 0.69, and a positive predictive value of 79% and negative predictive value of 57% with the time-signal intensity curve method (p = 0.003, 0.004, 0.008). The diagnostic consistency of the three radiologists was shown by the κ-value, 0.857 (p<0.001) with the method based on the time-signal intensity curve and 0.826 (p<0.001) with the method of the kinetic model.

**Conclusions:**

According to the statistic results based on the 46 lesions, the kinetic modeling curve method showed higher sensitivity, specificity, positive and negative predictive values as compared with the time-signal intensity curve method in lesion classification.

## Introduction

Breast cancer is a common cancer in women worldwide. Currently, noninvasive imaging techniques used to screen and diagnose the disease include mammography, sonography, magnetic resonance imaging (MRI) and tomosynthesis [1–3]. MRI provides not only morphologic information regarding lesions, but also information regarding the functional characteristics of lesions, such as tissue perfusion and enhancement kinetics. In addition, the image quality of MRI is not significantly impaired by dense tissue, which leads to a higher sensitivity in the detection of breast lesions [4–8]. MRI can better characterize lesion characteristics and the information about lesion vascularity can be obtained with the use of contrast medium [9]. Therefore, the trend of using MRI for the diagnosis of breast cancer is rapidly increasing. Recently, dynamic contrast-enhanced MRI (DCE-MRI), an MRI technique that employs a time-signal intensity curve, also known as a kinetic curve, obtained by repeated MRI scans after contrast agent injection has emerged as a useful tool for the screening of breast cancer due to its high detection sensitivity [10–12]. In this technique, the shape of the time-signal intensity curves usually classified as persistent enhancing (Type 1), plateau (Type 2), or washout (Type 3). Type 1, a persistent enhancing curve, which demonstrates a persistent increase in signal intensity after contrast injection, is correlated with benign lesions. Type 2, a plateau curve, which shows a slow or rapid increase in the beginning and then exhibits a sharp bend and plateau, is indicative of malignancy. Type 3, a washout curve, which has a drop-off in signal intensity with time after a rapid initial rise in the beginning, is known to be relatively specific for malignant lesions [8]. However, several studies have reported that time-signal intensity curves have a very high sensitivity but a relatively low specificity for the diagnosis of breast cancer [10,13–16]. Many studies have applied kinetic models to improve the diagnosis of breast lesions. Henderson *et al*. developed a technique by which blood flow, blood volume, and capillary permeability can be measured simultaneously in breast tumors [17]. Delille *et al*. demonstrated that the ratio of regional blood flow and blood volume quantified by a kinetic model can be used to distinguish a breast tumor from benign or normal tissue [18]. By using a two-compartment model, Brix *et al*. found that the ratio of capillary transfer coefficient/capillary plasma flow can be used to distinguish benign lesions from malignant tumors [19].

This study aimed to use a kinetic model with DCE-MRI to improve the specificity of the diagnosis of breast cancer as compared with the time-signal intensity curves method. We developed a quantitative measurement of breast lesions with DCE-MRI using a single-compartment model. This method first identifies a region of interest (ROI) that represents one compartment, and further analyzes the concentration of contrast and the flow from the inlet and outlet in the ROI. The diagnostic results using kinetic model were compared and correlated with the pathologic findings from the breast lesion specimens.

## Materials and Methods

### Patients

This retrospective study assessed patients with detectable breast lesions who underwent breast MRI in our hospital between 2011 and 2014 [S1 Dataset]. All cases contained definitive pathological diagnosis in their medical records. Totally, 43 female patients were included in this study, and data of 46 breast lesions were collected for analysis. The age distribution of the patients was between 30 and 65 years (45.23±8.16). The data used for this study were anonymously collected, and this study was approved by the Institutional Review Board of our hospital prior to the start of the study (CMUH103-REC3-052).

### MRI

All collected data were acquired using our 3T MR scanner in China Medical University Hospital, Taiwan (Signa HDxt, GE Healthcare, USA). Patients were asked to lie on the 8-channel breast coil with prone position when preforming the examination. The standard breast MRI protocol in our hospital included a T2-weighted fat-suppression sequence (short T1 inversion recovery, STIR), a regular T1-weighted fast spin echo (FSE) sequence without fat-suppression, and a dynamic contrast enhanced (DCE) T1-weighted fat-suppression sequence. All images were performed on transverse section (S1 Dataset). About the dynamic scan, we used a high spatial resolution 3D imaging technique, VIBRANT, which was provided by the vendor. This technique applies dual shim volumes which contains advantage of homogeneity during fat suppression. Such feature was important for the diagnosis of breast lesions as well as our further data analysis. An intravenous bolus injection of gadodiamide (0.1 mmol/kg body weight, Omniscan, GE Healthcare, Ireland) was carried out by using an automated injector with consistent rate of 2.6 ml/sec, we also applied another 20ml of saline wash. A total of eight continuous volumes were acquired for the DCE-MRI section, while the injection was started at the beginning of the second phase. The DCE-MRI employed the following parameters: slice number/volume = 78, slice thickness = 1.8 mm, field-of-view = 360×360 mm^2^, matrix size = 512×512 which was automatically interpolated into 1024x1024, repetition time (TR) = 10.35 ms, echo time (TE) = 4.25 ms, flip angle = 10, echo train length = 1.

Kinetic data were evaluated by placing an ROI as the compartment. ROIs should be placed into the area that exhibits strongest enhancement in each series. The ROIs were ensured to be within the lesion throughout the entire dynamic series. The change in the concentration of the contrast agent over time was calculated by analyzing the change in the gray-scale intensity in the corresponding area inside the ROI.

### Histological diagnosis

All patients received echo-guided biopsy or MRI-guided biopsy. All samples were embedded in paraffin and serially cut for immunohistochemical stains. Histological diagnoses were determined by an experienced pathologist.

### Kinetic model

The single-compartment model used in this study is based on Fick principle which was developed by Adolf Fick. The Fick principle has been applied in a variety of clinical situations, for example, the measurement of cardiac output. It is shown in the following equation [20]:

$$C2(t)=\frac{\Delta M}{F}-C1(t)$$ (1)

where *ΔM* (gray level/phase time) is the change in the gray level of the ROI from the DCE-MRI, *F* is the blood flow, and *C1* (gray level/pixel) and *C2* (gray level/pixel) are the average gray levels of the inlet and outlet blood flow in the compartment, respectively. By adding the *ΔM* value and replacing *C1* with the change in gray level of the arterial inlet in the ROI, the values of *C2* and F can be calculated by employing a least square fit. ROIs were within the lesion or the ascending aorta throughout the entire dynamic series by manual selection. The kinetic modeling curve can be generated from the change of the *C2* versus time.

### Statistical analysis

By using this kinetic model, each *C2* value corresponding to each image phase was obtained, and the plot of the *C2* values of the phase is called a kinetic modeling curve. All the kinetic modeling curves of the different lesions were analyzed by three experienced radiologists and classified as follows: Type 1: persistent enhancing type, Type 2: plateau type, Type 3: washout type. The results were then compared with the pathologic findings from the breast lesion histological diagnosis, and analysis was performed using a statistical analysis software, Statistical Product and Service Solutions (SPSS), to plot the receiver operating characteristic (ROC) curve and then calculate the area under the curve (AUC), sensitivity, specificity, positive predictive value (PPV) and negative predictive value (NPV). The results of the analysis were also compared with those obtained using the time-signal intensity curve method that is currently often used in the clinical setting. The consistency among the three radiologists was analyzed by Kappa (κ) value statistics.

## Results

**Fig 1:**
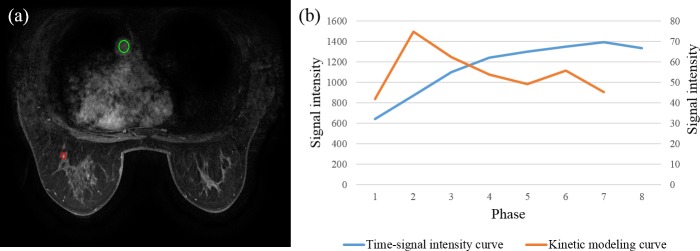
Application of the time-signal intensity curve and the kinetic curve in breast MRI analysis. (a) MRI of a 58-year-old female showed an invasive ductal carcinoma on the upper outer quadrant of the left breast. The green circle indicates the region of interest (ROI) that was used for estimating the change in the C1 value, and the red circle represents the ROI for measuring the ΔM. (b) The blue line is the time-signal intensity curve, and the orange line was generated from a plot of the C2 values to form a kinetic modeling curve.

**Fig 2:**
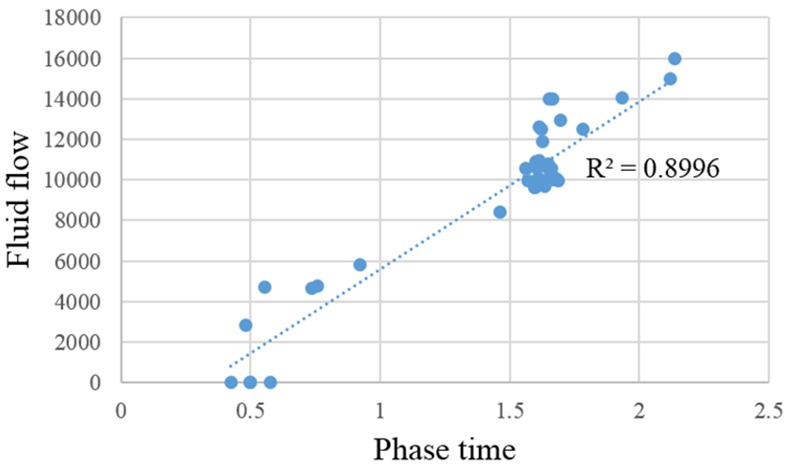
Linear regression analysis for the correlation between blood flow and phase time.

Of the 46 lesions analyzed in this study, 31 (67%) were malignant, which included 25 invasive ductal carcinoma (IDC), five ductal carcinoma in situ (DCIS), and one mucinous carcinoma. As shown in Fig 1, the plot of the calculated *C2* values to image phase (kinetic modeling curve, Fig 1B) can be determined based on the kinetic model. The findings of the three radiologists based on the time-signal intensity curve and the kinetic curve were compared. The consistency of the three radiologists was as shown in Table 1, the κ-value was 0.857 (*p*<0.001) with the method based on the time-signal intensity curve and 0.826 (*p*<0.001) with the method of the kinetic model. In addition, the correlation between blood flow and phase time was determined using linear regression, and the analysis showed that the squared correlation coefficient (*r*^2^) was 0.90 (Fig 2).

**Table 1:** The findings of the breast lesions from three radiologists based on the time-signal intensity curve and the kinetic curve. The sensitivity (SN), specificity (SP), area under the receiver operating characteristic curve (AUC), positive predictive value (PPV), negative predictive value (NPV), and kappa (κ) value are presented.

Reviewer	SN(%)	SP(%)	AUC	PPV(%)	NPV(%)	*p*-value	κ-value
Time-signal intensity curve 1	81	60	0.72	78	57	0.017	0.86
Time-signal intensity curve 2	81	53	0.68	81	60	0.052	
Time-signal intensity curve 3	77	53	0.67	77	53	0.068	
Kinetic modeling curve 1	81	67	0.77	83	63	0.003	0.83
Kinetic modeling curve 2	81	67	0.77	83	63	0.004	
Kinetic modeling curve 3	84	60	0.74	81	62	0.008	

**Fig 3:**
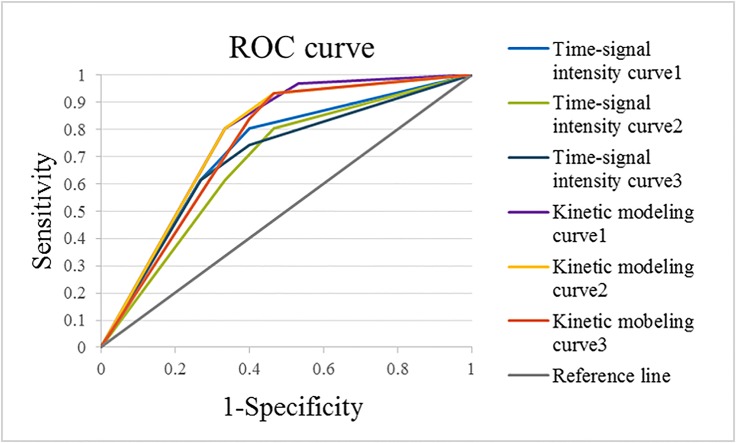
Comparison of the area under the ROC obtained by three radiologists using two different methods.

Using pathological examination of the breast biopsy as the standard, the results obtained using the time-signal intensity curve method showed an average sensitivity of 80±2%, a specificity of 55±4%, an area under the ROC of 0.69±0.01, and a PPV of 79±2% and NPV of 57±4%. On the other hand, when the kinetic model was used, the results showed an average sensitivity of 82±2%, a specificity of 65±4%, an area under the ROC curve of 0.76±0.01, and a PPV of 82±1% and NPV of 63±1% (Table 1). A comparison of the area under the ROC curve obtained by the different radiologists using different methods is shown in Fig 3. Using the time-signal intensity curve, for lesions categorized as of the persistent enhancing type (Type 1), the pathologic findings showed that the percentages of benign and malignant lesions were 57±4% and 43±4%, respectively. For those categorized as of the plateau type (Type 2), the percentages of benign and malignant lesions were 26±2% and 74±2%, respectively. And for lesions categorized as of the washout type (Type 3), the percentages of benign and malignant lesions were 20±2% and 80±2%, respectively. However, using the kinetic model, for samples categorized as Type 1 lesions, the percentages of benign and malignant lesions from pathologic findings were 83±5% and 17±5%, respectively. For those categorized as Type 2 lesions, the percentages of benign and malignant lesions were 32±6% and 68±6%, respectively. And for those categorized as Type 3 lesions, the percentages of benign and malignant lesions were 18±2% and 82±2%, respectively (Table 2).

**Table 2:** Percentages of benign and malignant Type 1 (persistent enhancing), Type 2 (plateau) and Type 3 (washout) lesions diagnosed by three radiologists based on the time-signal intensity curve and the kinetic curve.

	Type I		Type II		Type III	
Reviewer	Benign	Malignancy	Benign	Malignancy	Benign	Malignancy
Time-signal intensity curve 1	57%	43%	25%	75%	21%	79%
Time-signal intensity curve 2	60%	40%	25%	75%	17%	83%
Time-signal intensity curve 3	53%	47%	29%	71%	21%	79%
Kinetic modeling curve 1	80%	20%	33%	67%	17%	83%
Kinetic modeling curve 2	88%	12%	37%	63%	17%	83%
Kinetic modeling curve 3	80%	20%	25%	75%	20%	80%

## Discussion

Several studies have reported that the time-signal intensity curve has a high sensitivity but a low specificity in the diagnosis of benign and malignant breast lesions [10,13–16]. In the present study, we also employed the time-signal intensity curve to predict the malignant status of breast lesions, which showed an average sensitivity and specificity of 80% and 55%, respectively. Our result was similar to the diagnostic rate given by previous studies [10,13–16]. When the kinetic modeling curve was used, the average sensitivity and specificity were significantly increased to 82% and 65%, respectively. This result indicated that the kinetic modeling curve has a higher diagnostic accuracy as compared with the time-signal intensity curve (Table 1 and Fig 3). In addition, Table 2 demonstrates that the time-signal intensity curve method incorrectly categorized 43% of Type 1 lesions as malignant on average, while the kinetic model method only incorrectly categorized 17% of the cases. In addition, 20% of Type 3 lesions were incorrectly categorized as benign with the time-signal intensity curve method, while 18% of the cases were incorrectly categorized as benign with the kinetic model method. These results indicated that the kinetic modeling curve method has a higher diagnostic performance for malignancy in Type 1 and Type 3 lesions than the time-signal intensity curve method.

The kinetic model is a mathematical model widely used in quantifying medical imaging information. When used with dynamic imaging processing, this mathematical model can be applied to obtain dynamic (time-sequence) imaging quantitative data. Therefore, this kinetic model provides a time sequence of image changes caused by contrast medium that makes the diagnosis more effective. For example, cerebral blood flow could be quantified by using a model that tracks the translation of radioactive substance between the blood and tissue [21]. A similar model was also used on the analysis of radiolabeled material and successfully quantified the density of dopamine transporter in human brain. The dopamine is known as an important neurotransmitter that involves in many brain functions [22]. The kinetic model could also be applied to DCE computed tomography (DCE-CT) and DCE-MRI [23]. Several studies had developed different techniques to improve the application of kinetic model for diagnosis. For example, Sheiman and Sitek applied a single-compartment kinetic model in DCE-CT to quantify small-bowel perfusion and pancreatic perfusion in order to assess inflammatory bowel and pancreatic diseases [24,25]. For an application in DCE-MRI, Martin and coworkers used a simple kinetic model with contrast-enhanced first-pass perfusion MR imaging to analyze renal blood flow, and found that the measurements correlated well with phase-contrast imaging [26]. Roberts and colleagues demonstrated that dynamic contrast-enhanced MRI has the potential to be used for quantifying the difference in microvascular function between patients with and without Sjögren syndrome [27].

Several studies reported mathematical methods that improve the diagnostic accuracy of time-signal intensity curve [16, 28–31]. Shimauchi *et al*. developed the computer-aided method to quantify kinetic heterogeneity of breast lesions and the best AUC value was 0.69 for a 3T scanner [28]. In our study, all images were scanned by a 3T MR scanner. The average AUC value was 0.76 with kinetic modeling curve method. This result indicated that the kinetic modeling curve method was a potential tool to differentiate between malignant and benign breast lesions from MR imaging.

When kinetic model is used with MRI, in order to accurately estimate the parameters in the equation, the MRI signal intensity should be proportional to the concentration of the contrast agent. Martin and colleagues studied the correlation between MRI contrast agent concentration and MRI relative signal intensity [26]. They found that, if it is assumed that the gadolinium-chelate relaxivity is not dependent on tissue type, the MR signal intensity increases linearly below a contrast agent concentration of 3.5 mmol/L. In current study, the concentration of contrast agent used was 0.1 mmol/kg, which was within the range of linear correlation. Therefore, at the concentration we used, the kinetic model can minimize the T1 and T2* effects of imaged tissue and the influence of the imaging sequence properties, suggesting that adjustment was not necessary in this study because these factors did not affect the analysis within the linear range.

Baltzer and coworkers identified that non-mass lesions were the major cause of false-positives in time-signal intensity analysis of breast MRI [15]. In this study, 20% of the samples were non-mass lesions (κ = 0.817), and the false-positive rate of these non-mass lesions was 35% with time-signal intensity analysis. The false-positive rate increased to 63% when the kinetic model was used, which indicated that non-mass lesions were also the main reason for the high false-positive rate. To overcome this issue, it has been suggested that dynamic bilateral imaging and the distribution of non-mass lesions enhancement should be taken into consideration during the analysis, which can reduce false-positive diagnoses [8].

In the correlation analysis using linear regression, the blood flow obtained from the kinetic model was proportional to the MRI phase time (*r*^2^ = 0.90, Fig 2). This indicated that the estimated blood flow was in a reliable range. However, individual differences might still exist and may have affected the diagnostic findings. In addition, this study used a single-compartment model based on Fick principle, while Kety and Schmidt attempted to improve the model based on Fick principle and created a new Kety-Schmidt method [32]. This Kety-Schmidt kinetic model has more complete assumptions and has been successfully applied to DEC-MRI [26]. Moreover, many researchers have also used two-compartment kinetic models in MRI analysis. For example, Liu *et al*. defined the blood and liver as two different compartments, and evaluated the reticuloendothelial system function using MRI [33]. We are undertaking an investigation into whether moving from single-compartment to two-compartment modeling can increase the diagnostic accuracy of breast lesions.

In conclusion, MR images provide various kinds of information for the diagnosis of breast lesions, which include morphology, enhancement characteristics and kinetic analysis. Each of them provides useful, irreplaceable values to the diagnosis. The aim of this study was to improve the kinetic analysis but not to provide a replacement of diagnostic standard. Currently, morphological images still play an important role in the diagnosis of breast lesions. In this study, we integrated a single-compartment kinetic model and DCE-MRI to generate a kinetic modeling curve to improve the diagnostic accuracy of breast lesions. In the 46 lesions analyzed, the kinetic modeling curve method had higher sensitivity, specificity, positive and negative predictive values as compared with the time-signal intensity curve method. The results suggested that the kinetic modeling curve method could be a potential tool to differentiate benign from malignant breast lesions from MR imaging. Although this study remains limitation of small sample size, the results should qualify sufficient reliability. In the future, we will continue the data enrollment to improve the credibility of our approach. Moreover, through analyzing the longitudinal follow-up data by using this kinetic model, it might also give us some light about its possibility to become a predictor of disease progression.

## Supporting Information

S1 Dataset The raw data of MRI in this study. (RAR)
